# Perception of Social Cues of Danger in Autism Spectrum Disorders

**DOI:** 10.1371/journal.pone.0081206

**Published:** 2013-12-04

**Authors:** Nicole R. Zürcher, Ophélie Rogier, Jasmine Boshyan, Loyse Hippolyte, Britt Russo, Nanna Gillberg, Adam Helles, Torsten Ruest, Eric Lemonnier, Christopher Gillberg, Nouchine Hadjikhani

**Affiliations:** 1 Brain Mind Institute, EPFL, Lausanne, Switzerland; 2 Athinoula A. Martinos Center for Biomedical Imaging, Harvard Medical School, Massachusetts General Hospital, Charlestown, Massachusetts, United States of America; 3 Gillberg Centrum, University of Gothenburg, Gothenburg, Sweden; 4 Laboratoire de Neurosciences, Université de Brest, Brest, France; Birkbeck, University of London, United Kingdom

## Abstract

Intuitive grasping of the meaning of subtle social cues is particularly affected in autism spectrum disorders (ASD). Despite their relevance in social communication, the effect of averted gaze in fearful faces in conveying a signal of environmental threat has not been investigated using real face stimuli in adults with ASD. Here, using functional MRI, we show that briefly presented fearful faces with averted gaze, previously shown to be a strong communicative signal of environmental danger, produce different patterns of brain activation than fearful faces with direct gaze in a group of 26 normally intelligent adults with ASD compared with 26 matched controls. While implicit cue of threat produces brain activation in attention, emotion processing and mental state attribution networks in controls, this effect is absent in individuals with ASD. Instead, individuals with ASD show activation in the subcortical face-processing system in response to direct eye contact. An effect of differences in looking behavior was excluded in a separate eye tracking experiment. Our data suggest that individuals with ASD are more sensitive to direct eye contact than to social signals of danger conveyed by averted fearful gaze.

## Introduction

Autism spectrum disorder (ASD) is a neurodevelopmental condition affecting more than 1% of children [1], [2], characterized by deficits in social interaction and communication as well as by the presence of restricted interests and repetitive behaviors [3]. Absence or impairment of social instinct has been proposed to lie at the core of ASD [4].

**Figure 1:**
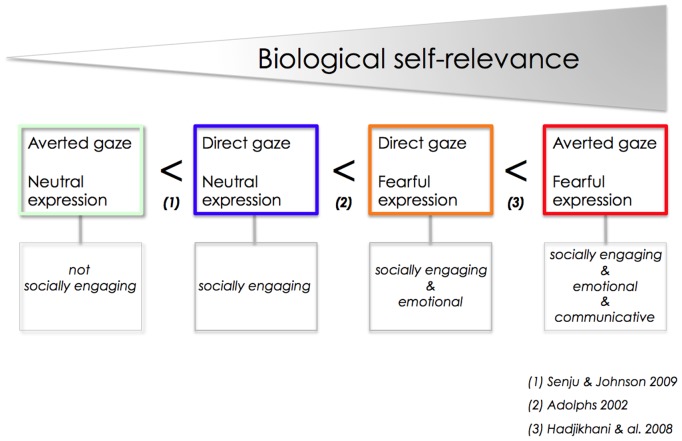
Face and gaze interactions depend on the degree of biological relevance conveyed. (1) For neutral faces, humans are more sensitive to direct gaze than averted gaze [67], as direct gaze reflects interest from a social partner and the beginning of a social exchange. (2) A face looking at us with a fearful expression is more arousing than a face with a neutral expression, due to the strong emotion it conveys [64]. (3) For fearful facial expressions, averted gaze is the most biologically self-relevant condition, with the social partner using non-verbal communicative cues to alert us to potential environmental danger [30].

Social observation is an efficient way to learn about potential harmful situations in the environment [5], [6], and evolutionary-old fear mechanisms are automatically engaged when typical individuals observe others showing signs of fear-related distress. Fearful expression and gaze direction are directly linked with biological self-relevance (Figure 1). In typical individuals, averted gaze in a fearful face is detected faster [7], rated as more intense than the same fearful expression with a direct gaze [8], and leads to automatic/reflexive gaze shifts [9]. Studies have shown that individuals with ASD show atypical brain activation in response to fearful facial expressions [10]) and to gaze [11]–[14]. However, despite their relevance in social communication, fear and gaze direction interactions have not been investigated using real faces in adults with ASD.

Gaze perception produces activation of the intraparietal sulcus, the superior temporal sulcus (STS) and regions of the dorsal and ventral fronto-parietal attention networks [15]–[18]. Saliency is captured by several areas: the amygdala ensures automatic attention to threatening stimuli [19], allowing biologically self-relevant stimuli to be processed even when outside the current focus of attention (reviewed in [20]); the pulvinar nucleus of the thalamus, through its reciprocal connections with the amygdala [21] and the superior colliculus (SC) [22], [23] contributes to the selection of salient stimuli [24], [25]; finally, the SC, associated with covert and overt shifts of attention [26], along with the frontal eye fields is involved in saccadic eye movement generation. The interaction of emotion and gaze direction hence involves various social attention processes including reorientation of attention, emotion processing as well as attribution of thoughts and intentions [27].

Using a paradigm with briefly presented fearful faces with averted or direct gaze for which we previously showed that it leads to modulation of attention and emotion networks [28]–[31], we aimed to investigate the neural response to fearful averted as opposed to fearful direct gaze in young adults with ASD. This paradigm relies entirely on social observation and although no social interaction is involved, the grasping of the meaning of these stimuli is particularly relevant for ongoing social interactions and communication. While previous studies in ASD have mostly used emoticons or avatars, this fMRI study investigates brain modulation in response to social cues of potential environmental threat using real face stimuli. We hypothesized that individuals with ASD would fail to grasp the meaning of this social prompt and would not show activation in brain regions associated with social attention compared to typical control participants.

## Materials and Methods

### Participants

The protocol was approved by the Lausanne University Hospital Ethical Committee and all procedures followed the Declaration of Helsinki. After complete description of the study was given to the participants, written informed consent was obtained. Twenty-six high-functioning individuals with ASD were enrolled in the study, from three centers (Lausanne, Brest and Gothenburg). For comparison purposes, 26 typical control individuals (CON) with no history of psychiatric or neurological disorders were recruited in Lausanne. Four participants with ASD and 4 CON had to be excluded due to excessive movement (>3 mm) during data acquisition. Thus 22 participants with ASD (19 males, 27.6 years±7.7 (mean±SD)) and 22 CON participants (19 males, 23.7 years±5.9) were included in the final data analysis. Participants in the ASD group were diagnosed according to DSM-IV-TR criteria by experienced clinicians [3]. The Autism Diagnostic Observation Schedule (ADOS) and the Autism Diagnostic Interview-Revised (ADI-R) [32], [33] were conducted for 14 ASD participants and the Diagnosis of Social and Communication Disorder-10 (DISCO-10) [34] was used for the participants from Gothenburg. All participants met criteria for autism spectrum disorder according to the current DSM 5 criteria [35]. In addition, autism traits were assessed in all participants but one using the Autism Quotient (AQ) self-report questionnaire [36]. The ASD group scored significantly higher than the CON group (ASD: 28.1±7.0; CON: 13.0±4.1; *t*(41) = 8.6, *p*<0.001). Performance intelligence quotient (PIQ) was assessed using the Wechsler Non-verbal Scale or the Wechsler Abbreviated Scale of Intelligence [37], [38] and all participants had a PIQ in the normal range (ASD: 114±15; CON 112±8). ASD and CON-groups did not differ in terms of age, intelligence quotient or gender. All participants had normal or corrected to normal vision. None of the participants of the current study were enrolled in the previously published study [30].

### Stimuli and paradigm

The paradigm used in the current experiment has been previously described in [30]. The stimuli were taken from the NimStim Set of Facial Expressions database [39]. Eight greyscale fearful faces (4 females) were selected and their gaze direction was altered by changing the position of the iris so that the faces were looking downwards toward the left or right, without altering their head direction (for an example of the stimuli used, see Figure 1 in [30]. A central fixation cross (FIXATION) was presented for 1200 ms followed by a face stimulus briefly presented for 300 ms in the center of the screen. This ensured that the eye region of the face stimuli appeared where the fixation cross was previously located and that the participants would attend to the eye-region [40]. Faces were presented in 24-second alternating blocks: 8 blocks of stimuli with direct (DIRECT) gaze and 8 blocks with averted (AVERTED) gaze (to the right in half of the blocks, to the left in the other half). Participants were instructed to observe the images attentively, and to look at the fixation cross, while trying to feel what the faces they were observing expressed.

### MRI data acquisition

Imaging data were acquired on a 3T scanner (Siemens Tim Trio, Erlangen, Germany) using a 12-channel matrix coil at the Centre d′Imagerie BioMédicale at the Centre Hospitalier Universitaire Vaudois in Lausanne. Slices were automatically positioned using the online AutoAlign Head LS (Landmark Survey) from Siemens. T1-weighted high-resolution (1.0×1.0×1.0 mm^3^) structural images were obtained at the beginning of the session with a multi-echo magnetization-prepared rapid acquisition gradient echo (ME-MPRAGE) sequence (176 slices, FOV = 256, matrix size 256×256, echo time (TE1) = 1.64 ms, (TE2) = 3.5 ms, (TE3) = 5.36 ms (TE4) = 7.22 ms; repetition time (TR) = 2530 ms; flip angle = 7°). Whole brain T2*-weighted gradient echo-planar images (EPI) were collected during the presentation of the paradigm. This functional acquisition (45 or 47 AC-PC slices, FOV = 216, matrix size = 64×64, TE = 30 ms, TR = 3 s, slice thickness 3 mm, flip angle 90°) lasted 384 s.

### fMRI data preprocessing and analysis

Whole brain voxel-wise analyses were conducted using FEAT version 5.98 part of FSL (FMRIB Software Library). For each subject first-level general linear model (GLM) analyses were conducted for the contrast averted vs. direct fearful gaze. Motion-correction was conducted using MCFLIRT and the motion parameters were added as nuisance parameters to the model. FSL's motion outlier detection program was used to identify residual outlier timepoints, which were included as additional confound variables in the GLM. Spatial smoothing using a Gaussian kernel of 8 mm, grand mean intensity normalization and highpass temporal filtering with sigma = 50.0 s were applied. Brain extraction of high-resolution anatomical images was carried out using Christian Gaser's VBM8 toolbox for SPM8 [41] and fed into FEAT. Subject-level analyses for the contrast AVERTED>DIRECT and DIRECT>AVERTED were performed using FILM. Non-linear Registration to the MNI template was carried out using the tool FNIRT. Group-level analyses were conducted using mixed effects with FLAME 1 and 2, allowing inference about the population from which the individuals were drawn. FSL's randomise was used to perform a permutation-based nonparametric statistical between-group (CON vs. ASD) analysis (n permutation = 10,000) using threshold-free cluster enhancement (TFCE). P values were family-wise error (FWE) corrected (*p*_FWE_<0.05). Local maxima where identified using *t* value maps as FWE-corrected clusters appeared large. A threshold of *t*>3.2 was chosen to control cluster size. Thus, only clusters which survived *p*_FWE_<0.05 and *t*>3.2 and contained at least 20 contiguous voxels are reported. All coordinates refer to MNI standard space. For visualization, statistical corrected *p* value maps (*p*_FWE_<0.05) are displayed on the pial cortical surface of the FreeSurfer brain (fsaverage) template (htttp://surfer.nmr.mgh.harvard.edu). In an additional analysis, the contrast AVERTED>FIXATION and DIRECT>FIXATION were compared within and between groups to control for potential differences in activation of face processing areas for the two different gaze conditions.

### ROI analyses

Regions of interest (ROIs) were selected to analyze activation of the subcortical route, known to be involved in the detection of biologically relevant stimuli, and consisting of the thalamus, the amygdala and the SC. To avoid circularity, ROIs were defined by independent anatomical constraints. The thalamus and the amygdala were identified using the respective label within the 25% probability Harvard-Oxford subcortical atlas. The SC was selected following anatomical landmarks [42]. Standard space anatomical ROIs were mapped back to subject space. Subsequently, for each ROI, mean percentage BOLD signal change within that ROI was extracted from the contrast of parameter estimate at the subject-level using FSL's Featquery. For each ROI Mann-Whitney U-test were conducted to assess differences between groups.

### Eye-tracking

To control for potential between-group differences in looking behavior, we conducted an eye-tracking study on a separate day after the fMRI experiment in a subset of the participants. Nineteen ASD and 14 CON participated in this experiment, but 3 ASD had to be excluded due to insufficient data (unsuccessful calibration or poor tracking quality). Data analysis was therefore conducted on 16 ASD and 14 CON.

### Data collection and analysis

Eye-tracking data was collected using a T120 eye-tracking system running Tobii Studio (TOBII Technology, Sweden). Participants sat comfortably 60–65 cm away from a 17-inch flat screen in a dimly lit room. Corneal reflection was measured for both eyes with infrared light sources and cameras, integrated in the monitor. A 9-point calibration was run prior to the experiment and data were recorded at 60 Hz. The same stimuli as those used in the fMRI were presented for the same amount of time as in the fMRI experiment (300 ms), preceded by a fixation cross (1200 ms). Areas of interest (AOI) were drawn for the eye region, the face and the computer screen. The eye region consisted of one rectangle covering both eyes and the bridge of the nose between the eyes. One large oval was used as AOI for the face. The total time spent looking at those areas was measured using Tobii Studio v.3.0.2. Eye fixations were determined using the criterion of eye position remaining within a 35-pixel area for a time greater than 80 ms. Analysis was conducted on absolute time spent looking at the eye region and at the face as well as on the ratio of time spent on eye region to time spent on the computer screen and time spent on face to time spent on computer screen. For each AOI total fixation duration differences in averted vs. direct gaze conditions were investigated within group using non-parametric Wilcoxon signed-rank tests. Between-group differences (CON vs. ASD) were assessed using two-tailed non-parametric Mann-Whitney U-tests.

## Results

### Eye-tracking results

No differences were found for the time spent on the *eyes* between gaze conditions or groups (CON: fear direct: 211 ms±23 (mean±SEM) and fear averted: 213 ms±24, ASD: fear direct: 190 ms±20 and fear averted: 195 ms±20, all *p*>0.05, *ns*.) and for the *ratio of time spent on the eyes* to time spent on the computer screen (CON: fear direct: 77.4%±0.8 (mean ± SEM) and fear averted: 78.0%±0.9, ASD: fear direct: 69.0%±0.7 and fear averted: 70.7%±0.7, all *p*>0.05, *ns*.). There were also no significant differences for the *face* region (CON: fear direct: 272 ms±2 and fear averted: 274 ms±2, ASD: fear direct: 261 ms±6 and fear averted: 270 ms±4, all *p*>0.05, *ns*.) and for the *ratio of time spent on the face* to time spent on the computer screen (CON: fear direct: 100.0%±0 and fear averted: 100.0%±0, ASD: fear direct: 95.6%±2.4 and fear averted: 98.9%±0.8, all *p*>0.05, *ns*.). This was expected given the chosen paradigm, designed to have participants look in the eye region (fixation cross presented where eye region of face would later appear), and the very short presentation time (300 ms).

### fMRI results

#### Within-group whole brain analysis - AVERTED>FIXATION and DIRECT>FIXATION

As expected based on the eye-tracking data, both ASD and CON showed increased activation in striate and extrastriate areas for direct and for averted gaze when compared to fixation. In particular, ASD and CON exhibited fusiform face area (FFA) activation in both conditions, indicating that participants in both groups were looking at the faces.

#### Within-group whole brain analysis - AVERTED>DIRECT

Within-group analysis showed that for AVERTED>DIRECT gaze, CON exhibited increased activation in several brain regions including the frontal eye fields, the intraparietal sulcus, the superior temporal gyrus, the FFA, the insula and the supramarginal gyrus (see Table 1) whereas ASD failed to demonstrate increased activation in any area for this contrast, even at a very liberal threshold (*p*<0.05, uncorrected).

**Table 1:** Within-group contrasts in CON.

	Region	Hemi	MNI			*t* value
			x	y	z	
**AVERTED>DIRECT**	Inferior temporal cortex	RH	58	−64	−16	5.96
		LH	−54	−62	−12	4.65
	Intraparietal sulcus	RH	14	−54	52	5.84
		LH	−18	−72	54	5.34
	Frontal Eye Field	RH	24	−10	48	5.55
		LH	−26	−10	50	5.1
	Precuneus	RH	9	−52	55	5.39
		LH	−10	−52	54	5.46
	Lateral Occipital Cortex	LH	−40	−78	12	5.22
		RH	42	−76	18	4.64
	Superior temporal gyrus anterior	RH	66	−10	0	5.16
		LH	−58	−10	−2	4.68
	Superior parietal cortex	LH	−26	−54	62	5.04
		RH	30	−46	64	4.35
	FFA	RH	42	−60	−16	4.97
		LH	−42	60	−20	3.42
	Superior temporal gyrus posterior	LH	−62	−42	14	4.82
		RH	66	−32	18	4.18
	Parieto-occipital sulcus	RH	24	−61	22	4.82
		LH	−18	−74	24	4.1
	Supramarginal cortex	RH	60	−32	44	4.72
		LH	−56	−32	44	4.11
	Mid Cingulate cortex	LH	−8	0	42	4.44
		RH	8	18	38	4.16
	Insula	RH	38	−12	−6	4.24
		LH	−42	−2	2	3.64
	Hippocampus	RH	20	−32	−6	4.07
	Inferior frontal gyrus, pars opercularis	LH	−56	2	6	3.65
**DIRECT>AVERTED**	*none*					

Brain regions for which CON showed increased activation for the contrast [AVERTED>DIRECT] gaze at *p*_FWE_<0.05, *t*>3.2. CON did not show increased activation for [DIRECT>AVERTED] gaze.

#### Within-group whole brain analysis - DIRECT>AVERTED

For DIRECT>AVERTED gaze, CON did not show increased activation even at a very liberal threshold (*p*<0.05, uncorrected). ASD participants did not show increased activation for direct gaze at *p*_FWE_<0.05. However, at a more liberal threshold (*p*<0.01, uncorrected), ASD showed increased activation for direct fearful gaze compared to avert in areas of the subcortical route, including SC and thalamus (but not the amygdala), and in fronto-insular cortex, anterior cingulate, posterior cingulate/precuneus, and cerebellum. See Table 2.

**Table 2:** Within-group contrasts in ASD.

	Region	Hemi	MNI			*t* value
			x	y	z	
**AVERTED**>**DIRECT**	*none*					
**DIRECT>AVERTED**	Cerebellum right VI	RH	30	−42	−26	4.19
	Anterior fusiform	RH	40	−34	−20	3.89
	Dorsolateral prefrontal cortex	RH	36	40	32	3.83
	Inferior occipital cortex	RH	26	−86	−8	3.82
	Anterior cingulate cortex	RH	2	24	28	3.29
	Thalamus	LH	−2	−22	2	3.25
	Precuneus cortex	RH	14	−58	30	3.24
	Fronto-insular cortex	RH	30	30	−6	3.07
	Caudate	LH	−10	8	10	2.9
	Superior colliculus	RH	6	−36	−8	2.76
	Cingulate gyrus	RH	14	−42	32	2.63

Areas, which showed increased activation in CON participants for the contrast [DIRECT>AVERTED] at *p*<0.01. Individuals with ASD did not show increased activation for [AVERTED>DIRECT].

#### Between-group whole brain analysis - AVERTED>DIRECT

**Figure 2:**
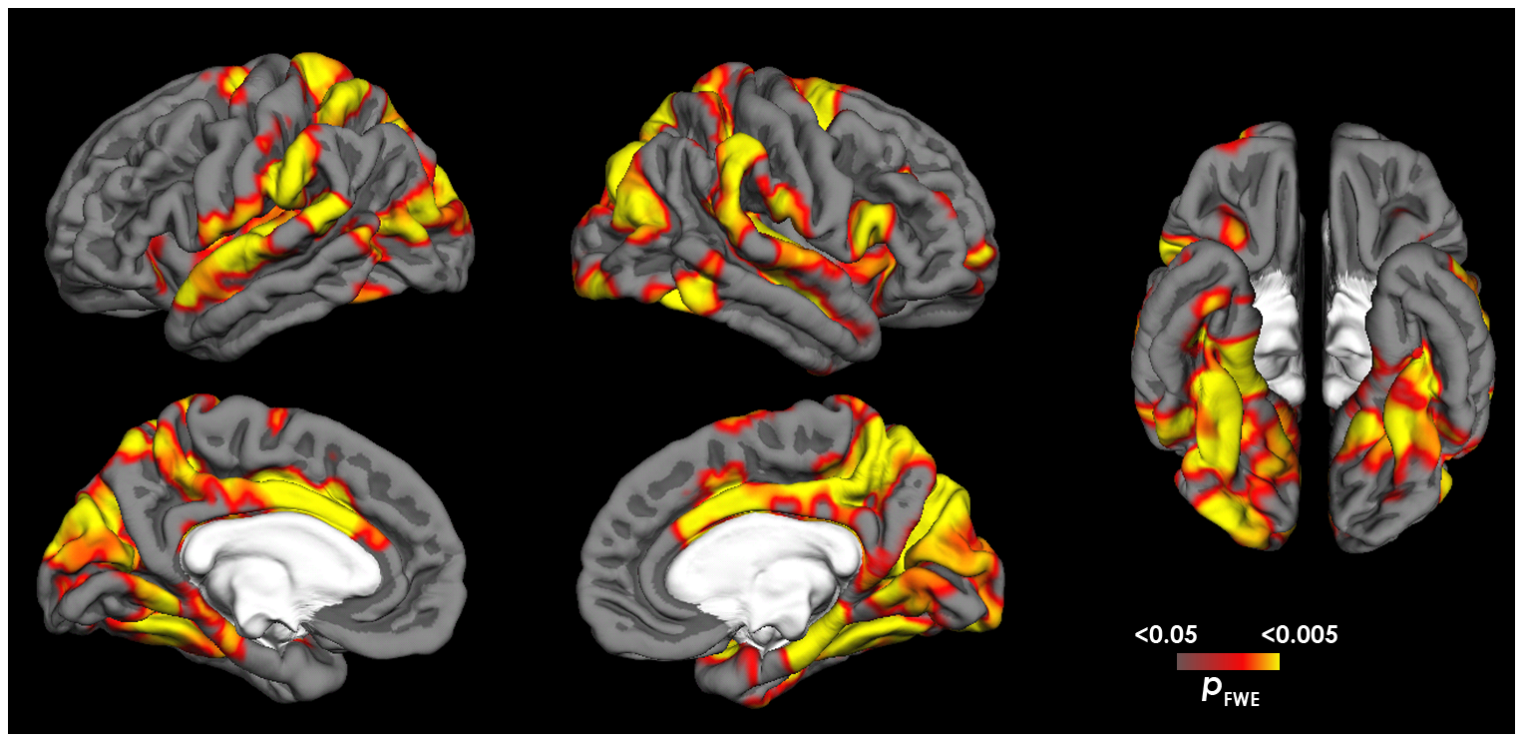
Cortical activation for averted gaze. Statistical maps of differences in fMRI activation for CON>ASD for the contrast averted>direct gaze (depicted in red to yellow). Group differences reflect increased activation for averted gaze in CON and lack of activation in ASD. Statistical maps are displayed on the lateral, medial and ventral views of both hemispheres, at *p*_FWE_ <0.05. The light grey mask covers subcortical regions in which activity cannot be expressed in surface rendering.

For the contrast AVERTED>DIRECT, CON showed increased activation compared to ASD in areas associated with gaze processing and attention including the intraparietal sulcus, superior parietal lobule, frontal eye fields, STS, superior temporal gyrus, temporo-parietal junction and supramarginal gyrus. CON also exhibited increased emotion processing in brain areas involved in emotion processing, including the anterior insula, anterior cingulate and posterior cingulate/precuneus cortex. In addition, increased activation was found for CON compared to ASD in the striate and extrastriate cortex, FFA, inferior occipital gyrus, inferior frontal gyrus, thalamus, hippocampus and cerebellum (*p*_FWE_<0.05, *t*>3.2, 20 contiguous voxels). See Figure 2, Table 3.

**Table 3:** Between-group contrasts: CON>ASD for [AVERTED>DIRECT].

	Region	Hemi	MNI			k	*t* value
			x	y	z		
**Gaze & Attention**	Anterior STS	RH	48	−6	−16	2127	3.93
		LH	−58	−8	−2	147	3.67
	Intraparietal sulcus	RH	18	−66	64	1190	4.31
		LH	−16	−70	54	130	3.64
	Frontal eye fields	RH	24	−4	66	1340	4.47
		LH	−26	−6	64	131	3.54
	Superior parietal lobule	RH	20	−46	68	1190	3.31
		LH	−24	−52	66	530	4.63
	Supramarginal gyrus	RH	60	−32	46	309	4.02
		LH	−56	−26	28	300	3.75
	Inferior frontal gyrus, pars opercularis	RH	54	20	14	31	3.38
	Dorsolateral prefrontal cortex	RH	40	48	30	183	4.39
	MT/V5	LH	−62	−60	10	28	3.73
**Emotion**	Anterior Insula	RH	42	16	−4	25	3.46
		LH	−42	10	−6	219	3.46
	Anterior cingulate	RH	6	18	38	1340	3.77
		LH	−6	2	38	1340	4.75
	Postcentral gyrus	RH	66	−16	28	65	3.69
		LH	−12	−40	50	54	3.93
	Hippocampus	RH	24	−22	−12	2127	4.18
		LH	−34	−18	−14	101	3.29
**Theory of Mind**	Posterior STS	RH	70	−36	4	24	3.73
	Superior temporal gyrus ant	RH	68	−8	0	34	3.71
	Temporo-parietal junction	RH	66	−30	28	58	3.38
	Temporal pole	RH	38	8	−24	2127	4.07
		LH	−52	14	−18	219	4.89
	Posterior cingulate cortex/Precuneus	RH	8	−38	46	1190	4.57
**Subcortical route**	Thalamus	RH	10	−18	8	58	3.77
	Superior colliculus	RH	8	−30	−8	2127	3.4
**Face processing**	Fusiform, FFA	RH	42	−60	−16	2127	4.3
	Anterior fusiform gyrus	RH	38	−36	−20	2127	4.22
		LH	−42	−32	−22	101	3.9
	Inferior occipital gyrus	RH	30	−88	−8	33	3.41
	Lingual gyrus	RH	8	−54	−2	91	4.06
**Visual** **processing**	Parieto-occipital sulcus	RH	16	−82	36	1371	5.11
	Inferior lateral occipital cortex	LH	−40	−82	10	64	3.66
**Other**	Cerebellum left crus I	LH	−36	−58	−40	115	3.79

Brain regions for which CON participants showed more activation than individuals with ASD for the contrast AVERTED>DIRECT, at *p*_FWE_<0.05, *t*>3.2.

### *A priori* ROI analysis

**Figure 3:**
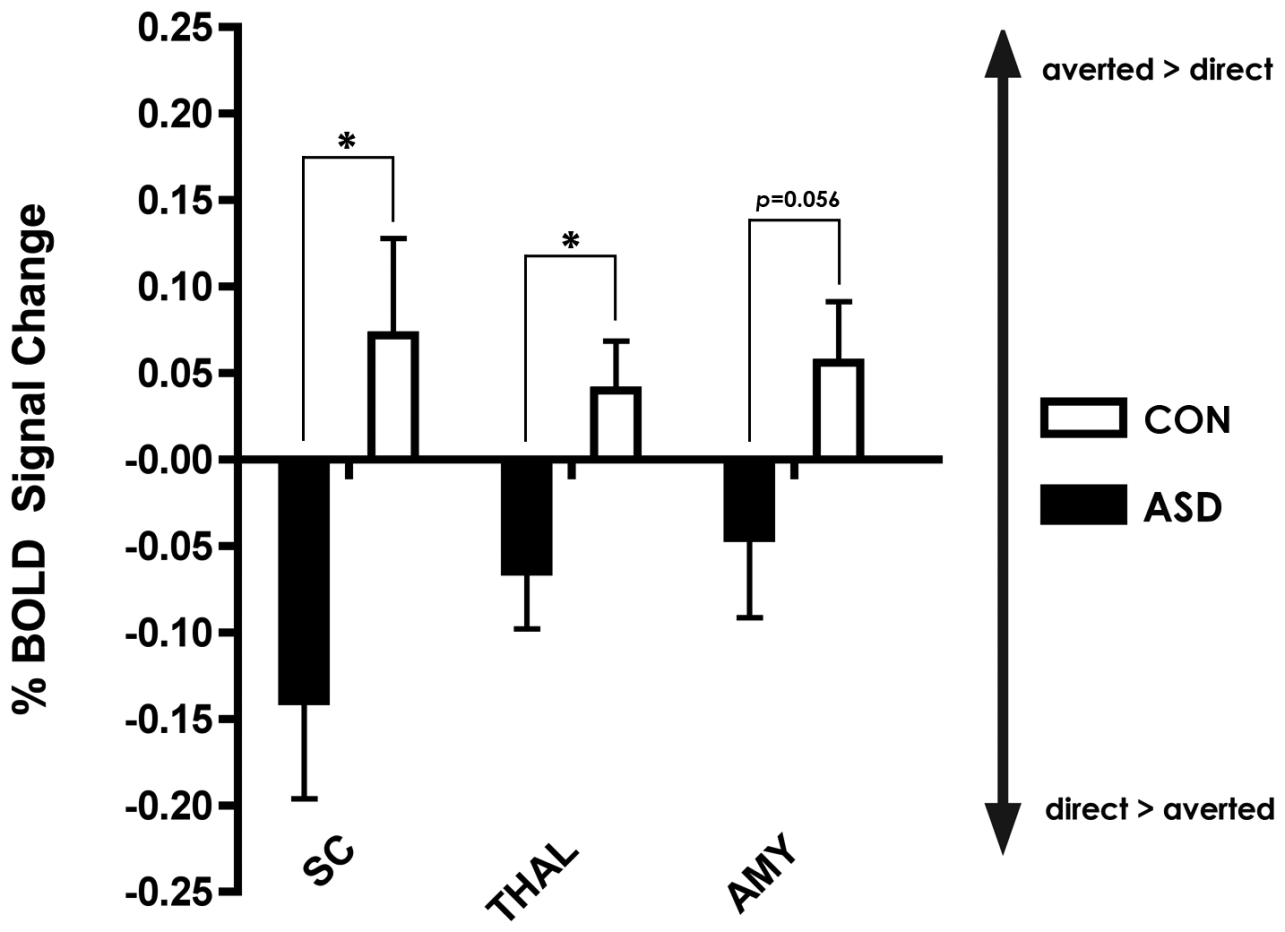
Region of interest analysis. Percent BOLD signal change (± SEM), for averted vs. direct gaze in selected subcortical ROIs. The thalamus (THAL) (*p* = 0.01), and superior colliculus (SC) (*p* = 0.04) were significantly different between ASD and CON while a strong trend was found for the amygdala (AMY) (*p* = 0.056).

For all subcortical ROIs, values were numerically greater for CON for the contrast AVERTED>DIRECT, indicating activation in controls for averted gaze. In contrast, ROI values for ASD for the contrast AVERTED>DIRECT were always negative, indicating that ASD showed more activation for the direct gaze condition. Significant between-group differences were observed for the SC (*p* = 0.01) and the right thalamus (*p* = 0.04), and showed a strong trend towards significance in the left amygdala (*p* = 0.056). See Figure 3.

## Discussion

Previous studies in autism have mostly investigated gaze and facial expression separately, leaving aside their interactive effects. Here, by combining fearful expression with different gaze directions, we demonstrate that the observation of social cues implicitly indicating the presence of a danger does not result in activation of brain areas involved in gaze perception, attention, emotion processing and mental state attribution in adults with ASD.

In our study, ASD participants failed to show typical activation in the dorsal and ventral fronto-parietal attention networks for averted vs. direct gaze. The absence of activation of these top-down and bottom-up attention networks suggests the lack of intuitive grasping of the biological relevance of the gaze cue and the absence of spontaneous reorientation. While studies using emoticons and studies using neutral faces have previously shown reflexive orienting in response to eye gaze cues in ASD [43], [44], the present study is to our knowledge the first to address the perception of the meaning of an emotional and social cue using real faces with emotional expressions in adults with ASD.

In ASD, gaze following behavior is developmentally delayed, and joint attention deficits belong to the earliest markers of this disorder [45]–[48]. Deficits in joint attention, i.e. deficits in the ability to non-verbally coordinate attention between individuals in order to share information regarding the environment, remain present in adults with ASD. Individuals with ASD do not spontaneously react to joint attention cues in videos with avatars, still emoticons with a neutral expression, or during live interactive video [12]–[14], [49]. The capacity to attribute mental states to others, also known as theory of mind (ToM), has been suggested to arise from joint attention [50] and individuals with ASD show deficits in ToM, as demonstrated by their decreased performance in the “reading the mind in the eyes” task [51], as well as by their lack of spontaneous mental state attribution to others [52] or to animated shapes [53]. In this study, the ASD group showed significantly less activation in areas associated with the attribution of thoughts, actions and intentions to others. Notably, we observed absence of modulation in posterior STS in response to gaze cues, a finding previously reported in ASD [14], [49]. The STS is involved in biological motion and gaze perception [54] and abnormal STS activation has been repeatedly described in autism (for review see [55]).

Unlike typical individuals, ASD failed to show increased activation in the anterior insula for averted fearful gaze. The anterior insula, structurally connected with the posterior STS through the superior longitudinal fasciculus, is sensitive to the social significance of eye gaze [56]. The insular cortex has been associated with multiple functions, ranging from performance monitoring [57] and attention to sensory and sensorimotor processing [58], and the activation in the ventral part of the anterior insula observed in controls is likely related to socio-emotional processing [58], [59], that is absent in ASD. The anterior cingulate cortex (ACC), involved in appraisal and regulation of negative emotion [60] was also significantly less activated in ASD.

Increased activation of the FFA for averted vs. direct gaze was observed in controls but not in ASD participants (although both groups showed FFA activation in both avert and direct conditions compared with fixation). The importance of the eye region in driving FFA activation has been shown in previous studies [61], [62]. In the eye-tracking experiment, both groups spent the same amount of time looking at the eye region in both conditions, strongly suggesting that different fixation times on the eye region are not the cause of the observed difference between groups in the FFA for averted vs. direct gaze. Instead, a more likely explanation is that participants with ASD, not grasping the increased emotional meaning of the averted gaze stimulus in the fearful face (a phenomenon previously reported in typical individuals [30] and linked with both attentional and emotional processes [63]), fail to modulate FFA activation in response to this biologically-relevant cue.

The detection of threat-related facial expressions and the ability to quickly read gaze direction play a central role for adaptive responses. Based on the literature, we propose a conceptual scheme emphasizing that the combination of facial expression and gaze direction are directly linked with biological self-relevance (See Figure 1). In neutral expressions (1), direct gaze leads to more activation than averted gaze, as direct gaze represents a desire to engage in a social interaction. Direct gaze associated with a fearful emotion (2), leads to more activation than neutral direct gaze (reviewed in [64]. Even more activation is observed for briefly presented fearful faces with averted gaze (3). Averted gaze in a fearful face is biologically self-relevant, and leads to shorter reaction times and increased amygdala activation in typical individuals [7], [28], [30], [31], [65].

**Figure 4:**
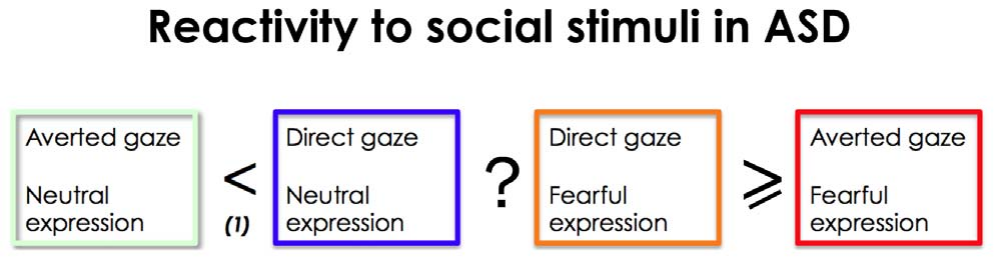
Atypical reactivity to social stimuli in ASD. Individuals with ASD show increased response to direct as opposed to averted gaze ((1) - Kylliainen 2006) but show atypical eye contact. While deficits in fearful face processing have been described in ASD, no study to our knowledge has specifically investigated fearful vs. neutral faces and it is unclear if individuals with ASD would show more activation in response to direct fearful gaze as opposed to direct neutral gaze. Finally, unlike controls, individuals with ASD do not show more activation for fearful averted gaze.

Figure 4 summarizes the findings for the processing of gaze in neutral and fearful faces in ASD. For *neutral* facial expression, individuals with ASD as well as controls show increased activation in response to direct neutral gaze as opposed to averted neutral gaze [66], [67]. Recent data show that this process is supported by the subcortical route, as amygdala activation for neutral direct gaze has been documented in a cortically blind patient [68]. The influence of direct gaze on behavior is referred to as “eye contact effect” reflecting the fact that perceived eye contact in others modulates cognitive processes (reviewed in [67]) and drives activation of areas associated with social processing, including the FFA, STS, amygdala and medial prefrontal cortex. In a study investigating the perception of socially relevant facial expressions either self- or other-directed (as indicated by gaze direction), ventromedial prefrontal cortex and medial temporal lobe/amygdala were shown to play an important role [69]. In ASD atypical eye contact effect has been observed, reflecting altered processing of direct gaze [70]. Increased subcortical activation in response to direct gaze in fearful faces in ASD is in line with reports of atypical modulation of arousal in response to direct gaze in children with ASD reported by Kylliainen et al, who measured greater skin conductance in response to direct than averted gaze [66], increasing as a function of the degree of eye openness [71].

Studies investigating modulation by *emotion* in direct gaze have shown diminished modulation of the face-processing network in ASD [72], [73], and a study conducted in adolescents with ASD reported that brain activations do not differ between averted and direct gaze in negative (anger and fear combined) facial emotions [74]. However, the results of this latter study do not allow to specifically draw conclusions about the interaction of gaze direction with a fearful facial expression as these two emotions were not analyzed separately. In addition, individuals with Asperger Syndrome do not have faster reaction times for fearful averted gaze, while controls show enhancement of joint attention by emotion [75].

To our knowledge, even though numerous studies have investigated the effect of fear vs. scrambled stimuli or the effect of various intensities of fear, no study has specifically compared fearful with neutral facial expressions (with direct gaze). Finally, in the current study, we show that individuals with ASD do not show increased activation for averted gaze in a fearful face. Instead, whole brain within-group and ROI analysis show increased activation of the subcortical face detection route in ASD for direct fearful gaze. This route, consisting of the SC, the thalamus and amygdala, [64], [76]–[79], is activated in typical individuals by direct eye contact in neutral faces [67], to a greater extent by direct gaze in a fearful face [64], [80] and to an even greater extent by an averted gaze in a briefly presented fearful face [29]–[31]. As shown by Senju and Johnson, the subcortical route may not appropriately modulate cortical and subcortical social brain networks in individuals with ASD [70], and the lack of top-down modulation together with decreased processing of mental and emotional states may therefore have lead to increased eye contact effect [81], [82].

Future studies should investigate the role of gaze direction in other emotional expressions and neutral faces.

## Conclusions

Using short stimulus presentation times, reflecting quick joint attention bids akin to how they occur in real life, we observed significant deficits in the activation of the distributed network of social attention in high-functioning individuals with ASD. Although both ASD and control participants looked similarly at the eye-region of the stimuli, networks involved in attention, gaze perception, emotion attribution and understanding of intentions were not engaged in individuals with ASD when processing social cues of danger. Instead participants with ASD showed hyper-activation of the subcortical route for direct gaze. This suggests that for individuals with ASD, eye contact with a fearful expression is more arousing than a fearful averted gaze signaling the potential presence of an environmental danger. These findings suggest that in early behavioral therapies, emphasis should be placed on association between eye-gaze cues and emotions, in order to specifically train the integration of these cues, thereby allowing young children with ASD to gain access to their social meaning.
